# Baicalein Ameliorates Aβ-Induced Memory Deficits and Neuronal Atrophy *via* Inhibition of PDE2 and PDE4

**DOI:** 10.3389/fphar.2021.794458

**Published:** 2021-12-13

**Authors:** Jing Shi, Yuanyuan Li, Yi Zhang, Jie Chen, Jianqing Gao, Tianyuan Zhang, Xiaoguang Shang, Xiangnan Zhang

**Affiliations:** ^1^ College of Pharmaceutical Sciences, Zhejiang University, Hangzhou, China; ^2^ School of Pharmaceutical Sciences, Institute of Materia Medica, Hangzhou Medical College, Hangzhou, China

**Keywords:** baicalein, memory and cognition, Alzheime’s disease, phosphodiesterase (PDE), neuroplasticity, neuroprotective effects

## Abstract

Inhibition of phosphodiesterase 2 and 4 (PDE2A and PDE4) increases the intracellular cAMP and/or cGMP levels, which may prevent Amyloid β 42 oligomers (Aβ) related cognitive impairment and dementias. Baicalein, one of natural flavones found in the root of Scutellaria baicalensis Georgi, has a wide range of pharmacological activities including antioxidant and anti-inflammatory effects. However, no studies suggest whether baicalein mediated anti-Alzheimer’s disease (AD) events involve PDEs subtypes-mediated neuroprotective pathways. The present study examined whether memory enhancing effects of baicalein on Aβ- induced cognitive impairment are related to regulating neuroplasticity via PDE2 and PDE4 subtypes dependent cAMP/cGMP neuroprotective pathway. The results suggested that microinjected of Aβ into CA1 of hippocampus induced cognitive and memory impairment in mice, as evidenced by decreased recognition index in the novel object recognition (NOR) task, impaired memory acquisition, retention and retrieval in the Morris water maze (MWM) and shuttle box tests. These effects were reversed by treatment with baicalein for 14 days. Moreover, Aβ-induced neuronal atrophy and decreased expression of two synaptic proteins, synaptophysin and PSD 95, were prevented by baicalein. The increased expression of PDE2A and PDE4 subtypes (PDE4A, PDE4B and PDE4D), and decreased levels of cAMP/cGMP, pCREB/CREB and BDNF induced by Aβ were also blocked by chronic treatment of baicalein for 14 days. These findings suggest that baicalein’s reversal of Aβ-induced memory and cognitive disorder may involve the regulation of neuronal remodeling *via* regulation of PDE2/PDE4 subtypes related cAMP/cGMP -pCREB-BDNF pathway.

## Introduction

For Alzheimer’s Disease (AD) is the most common pathogenesis of dementia with a global prevalence of 47.5 million. It is estimated that nearly 7 million people aged 85 and older will suffer from AD and related dementia by 2050 (Alzheimer’s, 2016). The AD has two typical pathological mechanisms: *β*-amyloid plaque (Aβ) deposition and neurofibrillary tangles of hyperphosphorylated tau (Briggs et al., 2016). Nevertheless, most treatments target toward Aβ and tau phosphorylation were proved not effective clinically (Oboudiyat et al., 2013). Thus, a growing interest has been raised in the development of effective therapeutics for the treatment of AD dementia, particularly multi-target botanicals from natural sources.

Baicalein is a critical flavone mainly found in the root of *Scutellaria baicalensis Georgi* (Sowndhararajan et al., 2017). Lines of studies demonstrate that baicalein exhibits anti-inflammatory, Antioxidant and antitumor properties (Liang et al., 2017; Zhu et al., 2020). It shows that baicalein improves long-term potentiation (LTP) in rat hippocampal slices and ameliorates hippocampus-dependent contextual fear memory in rats (Wang et al., 2011). More recently, there is evidence indicating, baicalein may server as a promising therapeutic agent for the treatment of neurotoxicity-mediated diseases (Sowndhararajam et al.,2017). However, few studies have reported that the interaction of baicalein with phosphodiesterases (PDEs) activity and the signaling pathway related to regulating neuroplasticity contributes to AD treatment.

*PDE2A* is a single gene that encodes phosphodiesterase 2 (PDE2); while PDE4 is encoded by four distinct genes and comprises four subtypes, PDE4A-4D (Gurney et al., 2019). Except for PDE4C, the other PDE4 subtypes (PDE4A, PDE4B and PDE4D) are widely expressed in brains and are related to *β*-amyloid (Aβ)-induced neuronal death, and learning and memory deficits (Xu et al., 2011). The brain distribution patterns of PDE4 subtypes are consistent with various implications for the central nervous system (CNS) dysfunction, such as cognitive deficits and dementia. Previous studies suggested that PDE2A and PDE4 expression was increased in brains of patients with AD and mouse models of AD, indicating that their involvement in AD pathology (Nabavi et al., 2019; Tibbo et al., 2019). Recent studies suggest that baicalein prevents Aβ-induced memory deficits by attenuating inflammatory response and neuronal apoptosis (Acquarone et al., 2019; Ji et al., 2020). However, whether the effects of baicalein on AD-related memory deficits are involved in regulation of PDE subtypes and the downstream signaling pathway remain unclear.

The present study aimed to explore the mechanisms by which baicalein protects against Aβ-induced memory impairment in mice. The results suggested that baicalein ameliorated Aβ-induced memory deficits and neuronal atrophy via downregulation of PDE2A and PDE4 subtypes’ expression and activation of the subsequent cAMP/cGMP dependent neuroprotective pathway.

## Materials and Methods

### Animals

ICR mice weighing from 22 to 30 g (2-3-month-old) were obtained from the Animal Center of Hangzhou Medical College (Hangzhou, China). They were adapted to the laboratory environment for 5–7 days before starting the experiment. In the animal center, mice were housed in a barrier system, which has specific pathogen-free conditions, controlled temperature (25 ± 1°C), and 12 h light/dark cycle. The animals had free access to water and food. The experimental procedures were performed following the National Institutes of Health Guide for Animal experimentation and the European Communities Council Directive of 24 November 1986 (86/609/EEC), which were approved by the Care and use of Laboratory Animals Committee of Hangzhou Medical College.

### Drugs and Treatment

**FIGURE 1:**
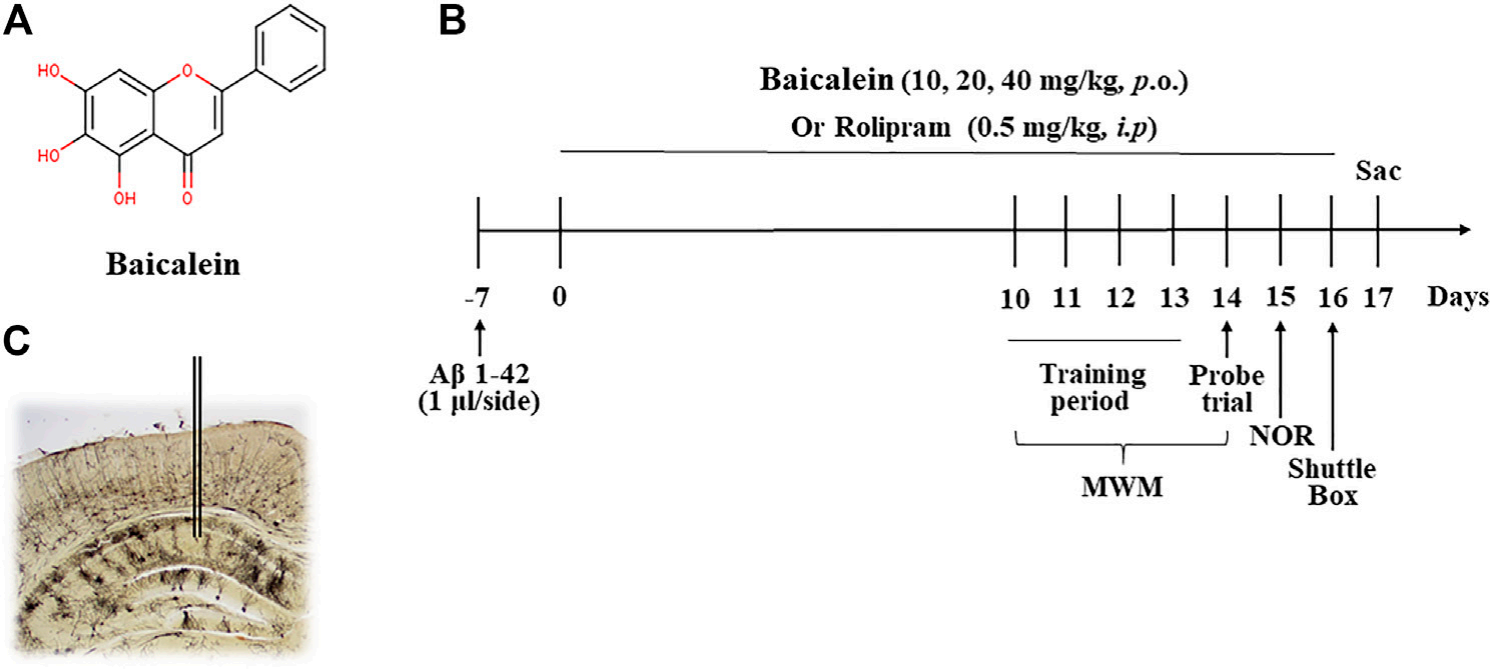
The chemical structure of baicalein **(A)**, treatment timeline and test order for mice treated with different doses of baicalein **(B)**, and **(C)** photomicrographs of representative cannula placements in the CA1 of hippocampus for treatment of Aβ oligomers were shown. MWM, Morris water maze; NOR, novel object recognition; Sac, sacrifice.

Aβ1-42 oligomers (Aβ) were ordered from R&D Systems-Hematology Group (Minneapolis, MN, United States), and were diluted in phosphate buffer solution (PBS, pH 7.4) to make the stock solution and stored at −70°C. The 100 μM of Aβ1-42 oligomers was microinjected into bilateral CA1 of hippocampus with 1 μl per site 14 days before drug treatment. Baicalein was purchased from Sigma-Aldrich Chemical Corporation (St. Louis, MO, United States) and was dissolved in dimethyl sulfoxide (DMSO). The concentration did not exceed 0.1% of the total volume in the working solution (The chemical structure of the baicalein shown in Figure 1A). Baicalein at 10, 20, and 40 mg/kg was continuously administered *via* gavage (i.g.,) for 14 days. Rabbit polyclonal antibodies directed against synaptophysin, PSD95, brain derived neurotrophic factor (BDNF) and beta-actin (actin) were bought from Santa Cruz Biotechnology (Santa Cruz, CA, United States). Rabbit polyclonal anti- bodies directed against cAMP response-element binding protein (CREB) and phospho-CREB (pCREB) were purchased from Cell Signaling Technology (Beverly, MA, United States). Horseradish peroxidase (HRP)-conjugated secondary antibodies were from Zymed (South San Francisco, CA, United States).

### Surgery and Experimental Schedule

Forty-six 2-3-month-old ICR mice were randomly divided into six groups (8 mice/per group): normal control, Aβ+vehicle, Aβ+10 mg/kg baicalein, Aβ+20 mg/kg baicalein, Aβ+40 mg/kg baicalein, and Aβ+0.5 mg/kg rolipram (as positive control group). The mice were anaesthetized with intraperitoneal (i.p.) administration of ketamine (100 mg/kg) and xylazine (6 mg/kg) before they were placed in a stereotaxic apparatus (Stoelting, Wood Dale, IL). Guide cannulae (22- gauge, 6 mm; Plastics One, United States) were implanted into bilateral CA1 subregions of the mice using the coordinates as described previously (Lehohla et al., 2001): (CA1: AP −1.5 mm from Bregma; ML ±1.2 mm from the midline; DV −1.5 mm from dura). The cannula was fixed to the skull with dental cement and then stainless stylets were inserted into the guide cannula for microinjection. The mice were put back in their home cage for recovery for at least 5 days. On day 6, Aβ42 oligomers were microinjected into bilateral CA1 of the hippocampus (1 μl/side) by an infusion pump. The cannula was kept in the microinjection site for an additional 2 min to avoid backflow. Seven days after Aβ42 oligomers microinjection, various doses of baicalein (Sigma-Aldrich, St. Louis, MO, United States, 10, 20, and 40 mg/kg via gavage, i.g.,) or the vehicle (0.1% DMSO), and rolipram (Sigma-Aldrich, United States, 0.5 mg/kg, *i.p.*), were treated once per day for 14 days. One hour after the last treatment on day 14, behavioral tests were conducted. After the behavioral tests on day 17, the mice were sacrificed (Figure 1B).

### Morris Water Maze

The procedure for carrying out the Morris Water Maze test was nearly identical to those followed by previous experiments (Xu et al., 2015). A water maze apparatus located in a well-illuminated room, consisting of a circular, plastic pool (1.5 m diameter × 0.5 m high), filled with opaque water (22 ± 1°C) with a platform 1 cm below the water surface was used. An automatic image acquisition system was connected with a behavior analysis system for tracking the learning and memory behavior. The water maze experiments containing acquisition trials and probe trials were performed. In the acquisition trial, mice were put in the center of four quadrants facing the wall individually to get familiar with the environment. The acquisition trial was carried out for 3 times per day separated by 20 min intervals for 4 days. A time to reach to the platform within 60 s was recorded as latency. The probe trial was performed after 4 days training with the objective of measuring spatial memory consolidation and retrieval of mice. Swimming time and the mice motion path were also recorded by the automatic video tracking system. The number of entries in the target quadrant, the percentage (%) of time spent in the target quadrant and latency to the target quadrant where the platform was previously located were determined 24 h after training.

### Novel Object Recognition Test

The novel object recognition test is performed to measure the object memory recognition ability of mice and the degree of scattered memory loss in mice similar to those of Alzheimer’s disease symptoms (Xu et al., 2015). A transparent open cube box (40 cm× 40 cm× 45 cm) was selected for the experiment which was divided into three stages, habituation, training and probe trials. In the habituation trial, all mice were allowed to freely explore the apparatus for 5 min. The training trial was carried out by placing an individual mouse in the center of the apparatus. The apparatus contained 2 fixed and odorless identical objects located in 2 diagonal corners. In the probe trial, mice were put back to the center of the same box; however, one of the familiar objects was replaced with a novel object. The ratio of time spent on new objects to the total time for exploring both novel and old objects was calculated as the discrimination index (DI). The total distance and the path mouse moves were also recorded.

### Shuttle Box Test

The shuttle box test was another approach to evaluate memory loss in rodents, as described previously (Saghaei et al., 2020). The shuttle box apparatus used in the experiment was a box which was divided equally into two small rooms, one bright room and one dark room. It contained a door opening in the middle connecting two rooms. Both rooms were equipped with incandescent lamps as light stimulus, and with a copper bar which can be electrified to provide electric shock stimulus. The habituation trial was carried out by placing all mice into the bright room and leaving an open door between the bright and dark room. All mice were allowed to move freely and familiarize themselves with the environment for 5 min. Furthermore, the time when the mice first entered the dark room without being stimulated by electric shock was recorded. The mice were then subjected electric shock for 20–30 times to acquire the ability of active avoidance conditioned response in the dark room in the training trial. Twenty-4 hours later, the probe trial was performed without electric stimulation; subsequently, the time when mice first entered into the dark room within 50s was recorded and analyzed by video tracking system.

### Golgi Staining Assay

The half brain of each mouse was rapidly processed in accordance with the instructions of a rapid Golgi staining kit (FD NeuroTechnologies, Ellicott city, MD) after the behavioral tests. The entire hippocampus (1.72–6.72 mm from the bregma) was cut into 100 μm potions of each slice (Xu et al., 2009). The procedure was followed by previously established methods in our lab that successfully stained hippocampal pyramidal cells (Xu et al., 2009). Sections were coded during processing and decoded on the completion of analysis. One of every ten slices was taken out for the Golgi staining assay, so a total of five slices of each mouse, i.e., 40 slices for each group, were analyzed. For the statistical analysis, cells were chosen based on the following criteria: the cell was located in the CA1 of hippocampus and relatively isolated from surrounding neurons. The average values of five pyramidal neurons from each mouse were treated as one sample, six samples (30 neurons totally) were analyzed in each group for morphological quantification. The number of dendrites and total dendritic length were quantified from a distance range of 50–400 µm from the soma (Vyas et al., 2002). To calculate the number of spines per 10 µm (spine density), the exact length of the dendritic segment was calculated, and the number of spines along the length was counted.

### Immunoblot Assay

After behavioral tests, animals were sacrificed, the half brain of each mouse was taken for immunoblot assay and the other half was prepared for Golgi staining test. The hippocampi from half brains were rapidly punched out and stored at -70°C. The samples were suspended in a solution containing 2% sodium dodecyl sulfate (SDS), 10% glycerol, 100 mM dithiothreitol, 0.01% (w/v) bromo- phenol blue and 60 mM Tris-HCl (pH 6.8) and sonicated for 10 s. Lysates were centrifuged at 12,000 g for 15 min at 4°C and the supernatant was collected for measurement of protein concentration. A sample consisting of 50 μg of protein was loaded into each lane and separated by 10% SDS-PAGE gel. After electrophoresis, the separated proteins were electrically transferred onto polyvinylidene difluoride membranes and were blocked in Tris-buffered saline with 0.1% Tween 20 (TBST) containing 5% non-fat dried milk for 1 h at room temperature. Subsequently, the membranes were incubated with anti-synaptophysin, anti-PSD95, anti-CREB, anti-pCREB at Ser133 or anti-BDNF primary antibody at a 1:1,000 dilution and shaken on a rotator at 4°C overnight. Following the washes with TBS containing 0.1% Tween-20 (TBS-T) for 5–6 min each, the membranes were incubated with 1:3,000 (synaptophysin, PSD95, CREB and pCREB) or 1:5,000 (BDNF) of anti-HRP-conjugated secondary antibody with 5% nonfat milk for 1 h at 20–22°C. The resulting antigen-antibody-peroxidase complexes were detected by enhanced chemiluminescent autoradiography (ECL kit; Amersham Pharmacia, Denver, CO, United States) and visualized by using the enhanced chemiluminescence method. Densitometer readings were used to quantitate the amount of protein in each group. The bands were subsequently analyzed densitometrically with Quantity One 4.2.3. software (Bio-Rad) (Xu et al., 2009).

### Statistical Analysis

All data were expressed as mean ± standard errors. Statistical comparisons were subjected to Student t test or one-way ANOVA in the behavioral tests. Differences were considered significant when *p* < 0.05.

## Results

### Baicalein Ameliorated Aβ42 Oligomers-Induced Sporadic Memory Deficits in Novel Object Recognition Test

**FIGURE 2:**
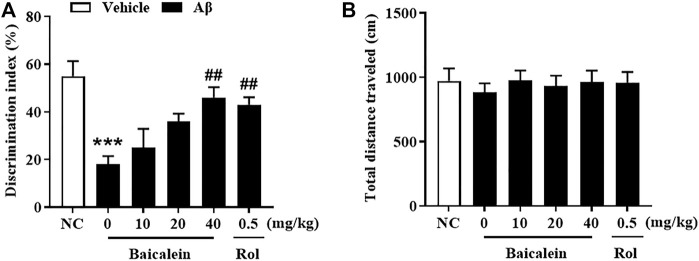
Baicalein improved cognitive function of Aβ-treated mice in the novel object recognition test. **(A)** The discrimination index was shown by the ratio of time spent exploring the novel object to the familiar one. **(B)** The total distance traveled of mice did not change. All values were expressed as mean ± SEM (*n* = 6). ^***^ *p* < 0.001 vs. normal control (NC) mice; ^##^ *p* < 0.01 vs. Aβ-treated mice.

The chemical structure of baicalein, experimental protocol, and the microinjection site of Aβ42 oligomers (CA1 of hippocampus) are shown in Figures 1A–C. As shown in Figure 2, the novel object recognition (NOR) test was performed to assess the episodic-like memory. Aβ42 oligomers induced significant memory impairment, as shown by a decreased discrimination index in 24 h after familiarization session when compared with that of control mice (Figure 2A, *p* < 0.001). Baicalein administration significantly increased exploration ability of mice to the novel object. One-way ANOVA analysis exhibited that baicalein improved memory retention and retrieval, in a dose-dependent manner, as shown by the increased discrimination index when compared with vehicle-treated Aβ42 group [Figure 2A, F _(5,42)_ = 5.82, *p* < 0.01]. PDE4 inhibitor rolipram also increased the discrimination index (*p* < 0.01). There was no significant changes in the total distance traveled among each group (Figure 2B), indicating that the performance differences did not result from the changes in overall activity.

### Baicalein Ameliorated Aβ42 Oligomers-Induced Spatial Memory Deficits in Morris Water Maze Test

**FIGURE 3:**
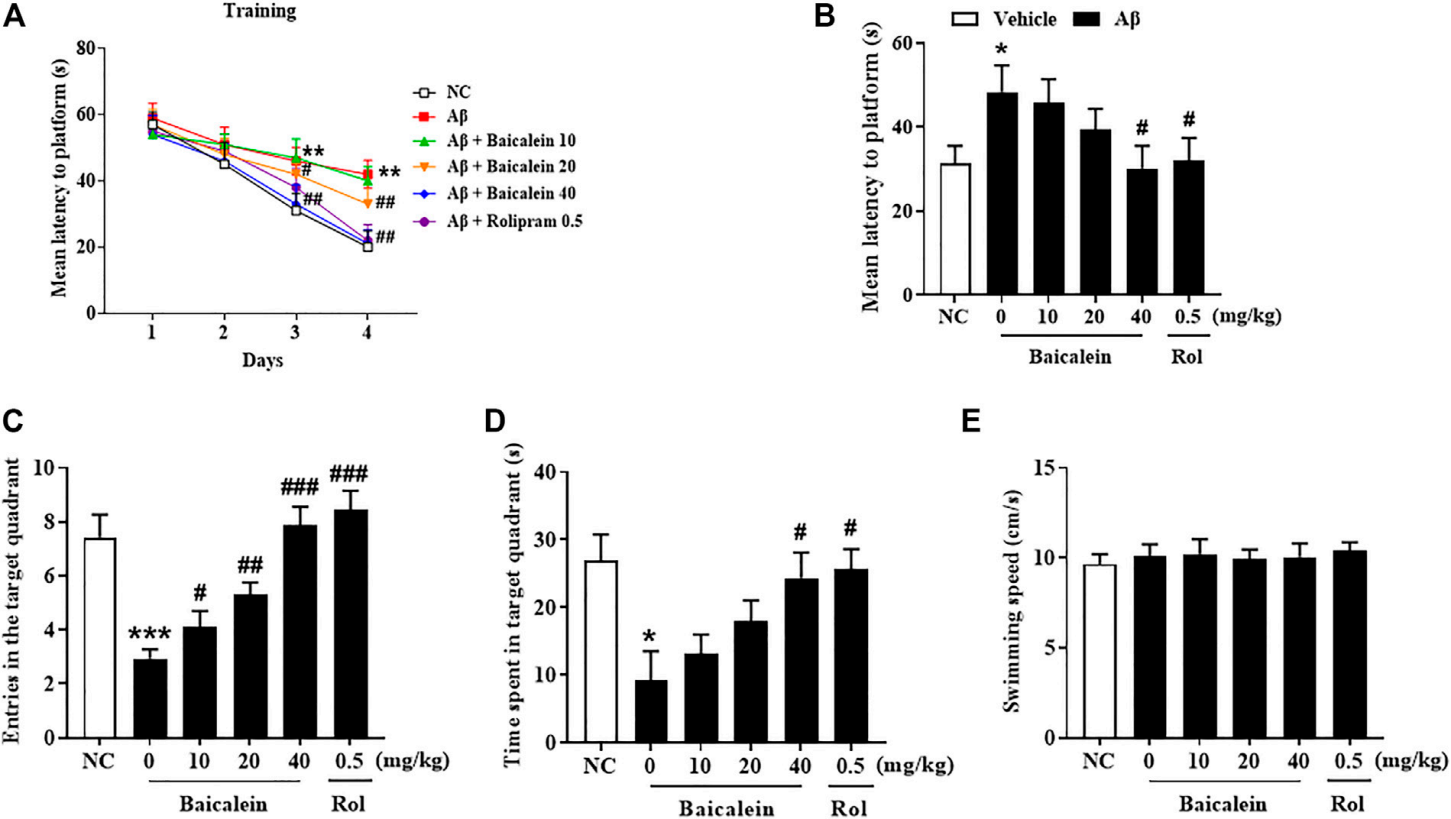
Baicalein improved memory acquisition and retention of Aβ-treated mice in the Morris water maze test. **(A)** The learning curve in the MWM test, **(B)** the mean latency to the platform, **(C)** the frequency of entering the target quadrant, **(D)** duration of time spent in the target quadrant, and **(E)** swimming speed were improved by baicalein treatment in Aβ-treated mice. All values were expressed as mean ± SEM (*n* = 6). ^*^ *p* < 0.05, ^**^ *p* < 0.01, ^***^ *p* < 0.001 vs. NC mice; ^#^ *p* < 0.05, ^##^ *p* < 0.01, ^##^ *p* < 0.001 vs. Aβ-treated mice.

Aβ42 oligomers altered learning of the MWM in mice as compared to the normal control group (Figure 3A). Mice treated with Aβ42 oligomers took significantly longer time to reach the platform on day 4 as compared to that of control group (Figures 3A,B, *p* < 0.01). This impairment was not present in those administered baicalein at 40 mg/kg via gavage (*p* < 0.01). Twenty-4 hours later, the platform was removed and the mice were tested in probe trials. Memory performance was worse in the Aβ42-treated mice in the 24 h test session after training; this was displayed by an increased latency to find the previous platform location, decreased the number of platforms crossing and time spent in target quadrant (Figure 3B, *p* < 0.05; Figure 3C, *p* < 0.001; Figure 3D, *p* < 0.05). Baicalein at 20 and 40 mg/kg reduced Aβ42-induced memory retention as shown by decreased time to find the previous platform location and increased time spent in the target quadrant (Figure 3B, *p* < 0.05; Figure 3D, *p* < 0.05). Furthermore, baicalein had an overall enhancing effect on platform crossing at both of the higher doses, 20 and 40 mg/kg (Figure 3C, *p*’s < 0.001) in the 24 h probe trial. There were also no significant changes in swimming speed among each group (Figure 3E). As the positive drug, rolipram had similar effects to those of baicalein at dose of 0.5 mg/kg in MWM test.

### Baicalein Ameliorated Aβ42 Oligomers-Induced Aversive Memory Deficits in Shuttle Box Test

**FIGURE 4:**
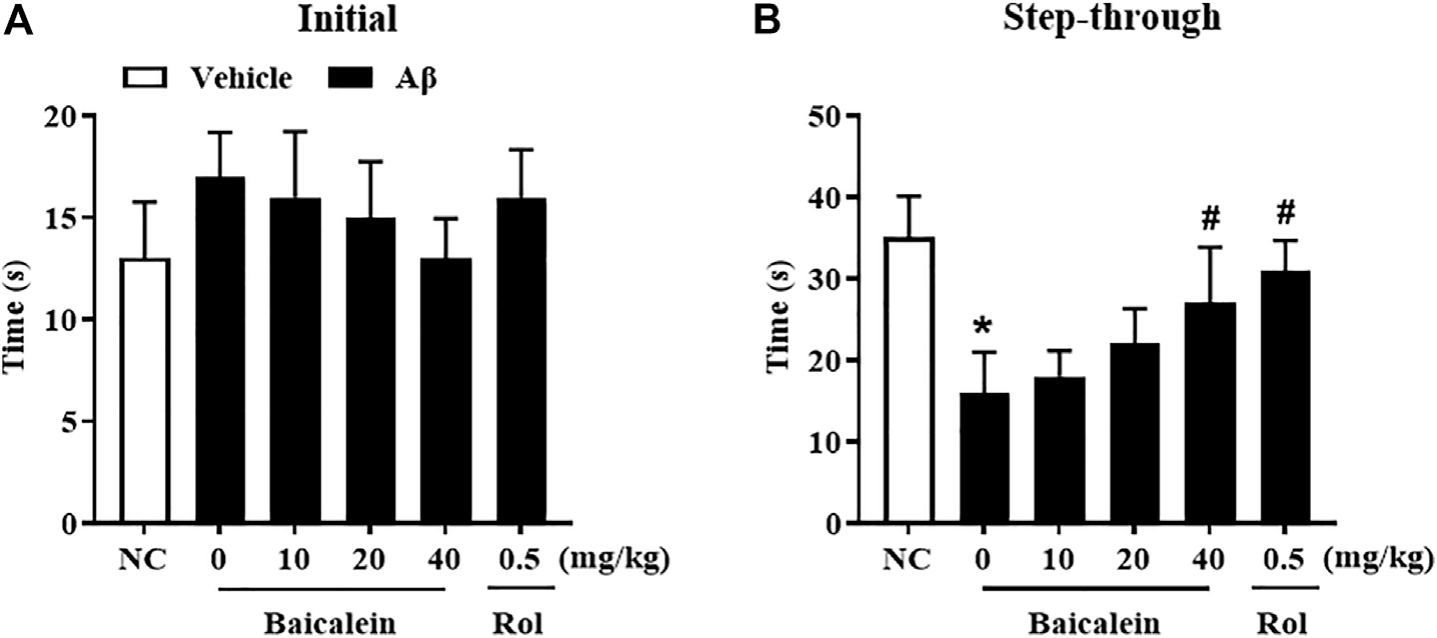
Baicalein ameliorated memory behavior of Aβ-treated mice in the shuttle box test. **(A)** Initial time to the black box without receiving stimulation, and **(B)** time to the black box after electric shock stimulation were ameliorated by baicalein treatment in Aβ-treated mice. All values were expressed as mean ± SEM (*n* = 6). ^*^ *p* < 0.05 vs. NC mice; ^#^ *p* < 0.05 vs. Aβ-treated mice.

Aβ42 oligomers-treated mice exhibited escape response deficits as shown in Figure 4. In the initial section, there has no significant changes in latency times among groups (Figure 4A). During the testing session, mice treated with Aβ42 oligomers took significantly less time to get into the dark box as compared to that of control group, although there was foot shock in the training session (Figure 4B, *p* < 0.05). Chronic baicalein treatment for 14 days significantly reduced the latency to cross the dark box, in these Aβ42 oligomers-treated mice, in a dose-dependent manner [Figure 4B,F _(5,42)_ = 9.27, *p* < 0.05].

### Baicalein Attenuated Aβ42 Oligomers-Induced Neuronal Atrophy in the CA1 of Hippocampus

**FIGURE 5:**
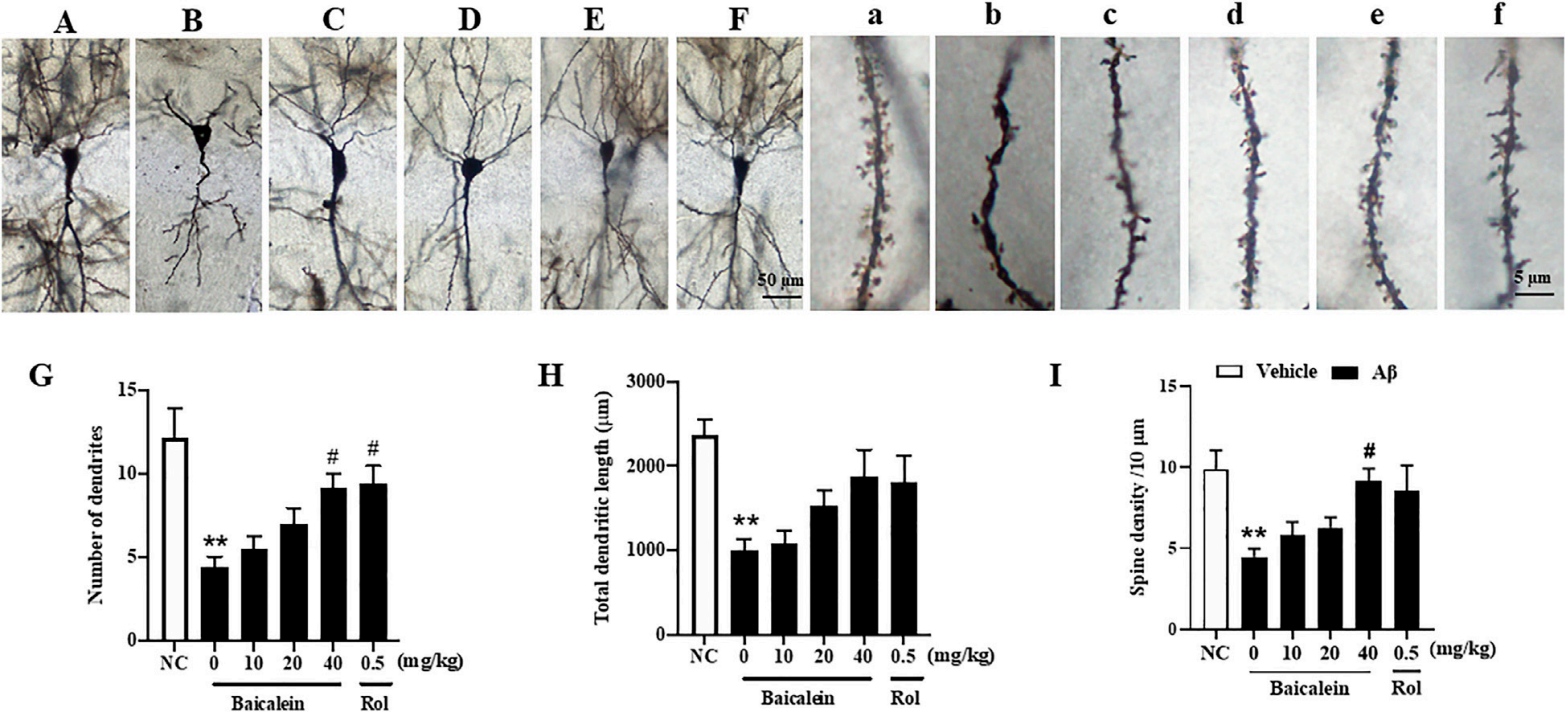
Baicalein improved hippocampal neuronal remodeling of Aβ-treated mice in the rapid Golgi staining assay. **(A–F)** and **(a–f)** Rapid Golgi staining showed increased neuroplasticity of the CA1 pyramidal neurons in the hippocampus (scale bars = 50 and 5 µm). **(G)** The number of dendrites, **(H)** total dendritic length, and **(I)** spine density per 10 μm were increased by baicalein treatment in Aβ-treated mice. All values were expressed as mean ± SEM (*n* = 6). ^**^ *p* < 0.01 vs. NC mice; ^#^ *p* < 0.05 vs. Aβ-treated mice.

In serial coronal sections of the hippocampus, pyramidal neuron’s atrophy was observed in the CA1 of hippocampus when mice were exposed to Aβ42 oligomers (Figures 5A–F; Figures 5a–f). The quantitative analysis showed that Aβ42 oligomers caused a significant decrease in the number of dendrites, total dendritic length, and spine density, 150–400 μm from the cell soma, as compared to vehicle-treated control groups (Figure 5, *p*’s < 0.01). Baicalein treatment for 2 weeks dose-dependently prevented Aβ- induced neuronal atrophy in the CA1 pyramidal neurons, by increasing number of dendrites [Figure 5G, F_(5, 42)_ = 8.19, *p* < 0.05] and spine density [Figure 5I, F_(5, 42)_ = 7.54, *p* < 0.05] in a dose-dependent manner. The tendency to increase the total dendritic length was also observed after treatment with baicalein (Figure 5H). The tendency to increase the total dendritic length and spine density were also observed. As the PDE4 inhibitor, rolipram increased the number of dendrites (*p* < 0.05). Therefore, these results suggest that baicalein protected hippocampal neuronal remodeling against Aβ insults.

### Baicalein Attenuated Aβ42 Oligomers-Induced Decreases in Synapse Associated Proteins’ Expression in the Hippocampus

**FIGURE 6:**
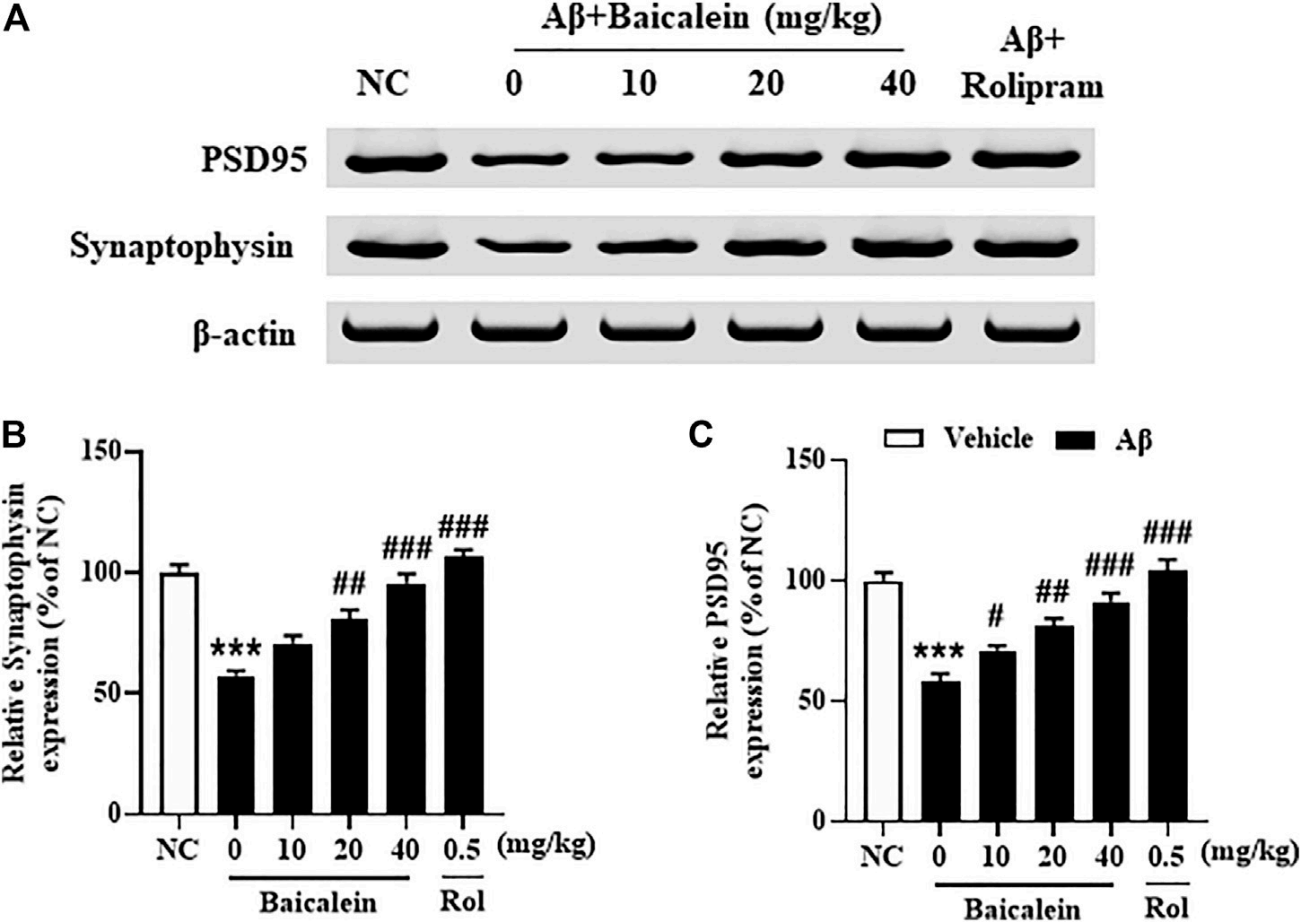
Baicalein rescued the expression of PSD95 and synaptophysin in the hippocampus of Aβ-treated mice. All values were expressed as mean ± SEM (*n* = 6). ***p < 0.001 vs. NC mice; #*p* < 0.05, ##*p* < 0.01, ##*p* < 0.001 vs. Aβ-treated mice.

Synaptic dysfunction contributes to neuronal atrophy and the deterioration of cognitive performance. The subsequent results showed that Aβ42 oligomers significantly decreased the expression of pre-synaptic protein synaptophysin (Figures 6A,B, *p* < 0.001) and post-synaptic protein PSD-95 (Figures 6A–C, *p*’s < 0.001) in the hippocampus of mice, as compared to vehicle-treated normal controls. Treatment with baicalein at 10, 20, and 40 mg/kg for 14 days significantly prevented the decreases of both synaptophysin and PSD-95 proteins in the hippocampus of Aβ mice, in a dose dependent manner, as compared to vehicle-treated Aβ mice (F _(5,42)_ = 10.32, *p* < 0.05; F _(5,42)_ = 9.84, *p* < 0.05).

### Baicalein Attenuated Aβ42 Oligomers-Induced Increased Expression of PDE2A and PDE4 Subtypes and the Downstream Proteins’ Expression in the Hippocampus

**FIGURE 7:**
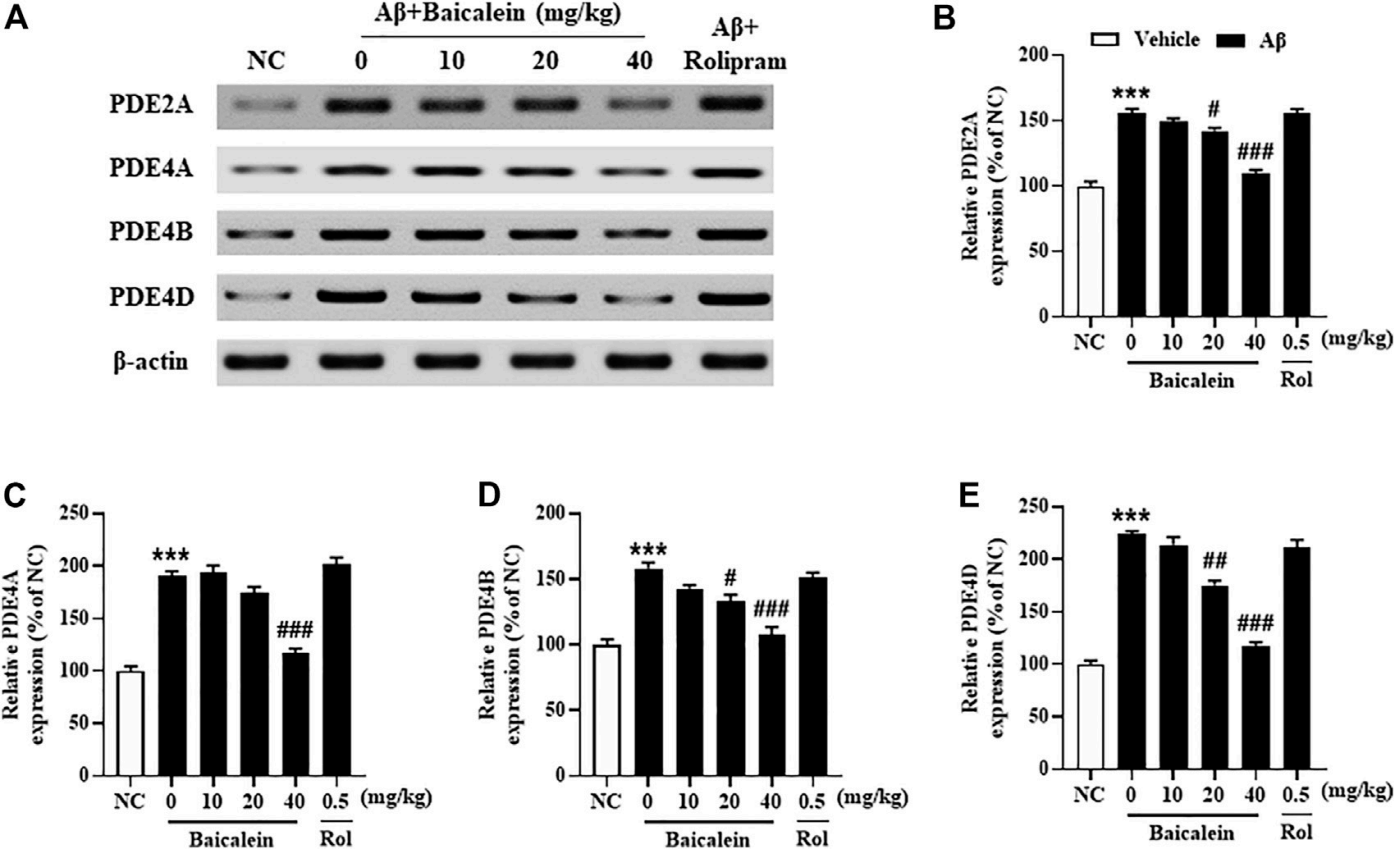
Baicalein decreased the expression of PDE2A, PDE4A, PDE4B, and PDE4D in the hippocampus of Aβ-treated mice. All values are expressed as mean ± SEM (*n* = 6). ***p < 0.001 vs. NC mice; #*p* < 0.05, ##*p* < 0.01, ##*p* < 0.001 vs. Aβ-treated mice.

**FIGURE 8:**
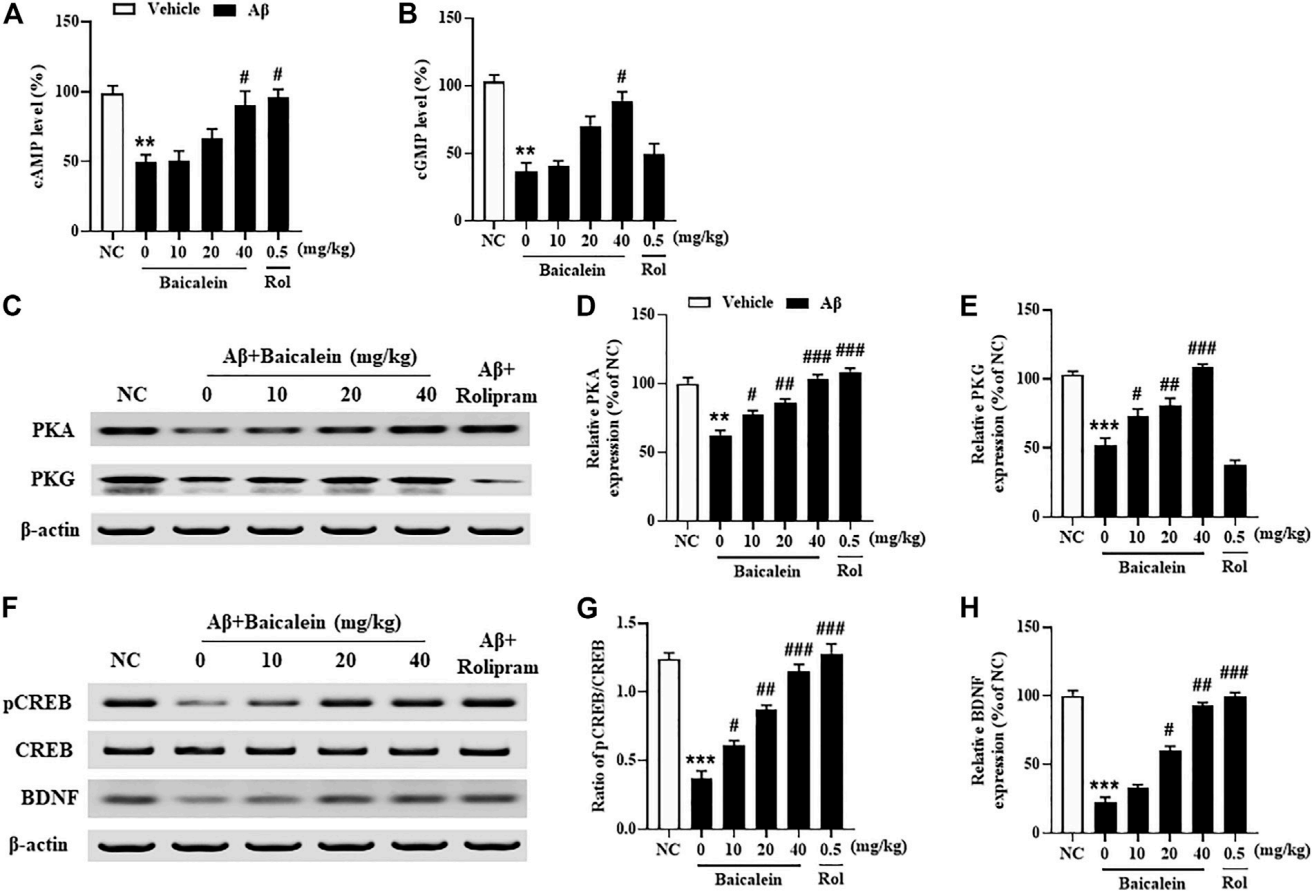
Baicalein increased cAMP- and cGMP-downstream proteins expression in the hippocampus of Aβ-treated mice. **(A,B)** The levels of cAMP/cGMP and **(C–E)** PKA and PKG, **(F,G)** the ratio of pCREB/CREB, and **(H)** the expression of BDNF were increased by baicalein treatment in Aβ-treated mice. All values were expressed as mean ± SEM (*n* = 6). ^**^ *p* < 0.01, ^***^ *p* < 0.001 vs. NC mice; ^#^ *p* < 0.05, ^##^ *p* < 0.01, ^###^ *p* < 0.001 vs. Aβ-treated mice.

Since the phosphodiesterase’s activity is closely related to Aβ induced neuronal atrophy by stimulating the hydrolysis of cAMP and cGMP, inhibition of PDEs contributes to cAMP/cGMP-dependent neuroprotective mechanism (Guo et al., 2017; Cui et al., 2019). The results showed that mice treated with Aβ42 oligomers increased the expression of PDE2A, PDE4A, PDE4B and PDE4D in the hippocampus (Figure 7, *p*’s < 0.001). However, chronically treatment with baicalein for 14 days significantly reduced these PDEs expression, i.e., PDE2A, PDE4A, PDE4B and PDE4D, in a dose-dependent manner [Figure 7B, F_(5, 42)_ = 9.14, *p* < 0.05; Figure 7C, F_(5, 42)_ = 11.61, *p* < 0.05; Figure 7D, F_(5, 42)_ = 8.87, *p* < 0.05; Figure 7E, F_(5, 42)_ = 9.14, *p* < 0.05]. These results were consistent with the subsequent ELISA assay, which suggested that cAMP and cGMP levels were increased after treatment with high dose of baicalein (40 mg/kg) (Figures 8A,B, *p*’s < 0.05). Rolipram at 0.5 mg/kg did not change Aβ42 oligomers-induced PDE4 subtypes’ expression due to its potential inhibition of PDE4 activity directly, but not affecting Aβ oligomers. This was supported by the further results that showed that cAMP levels were increased after treatment with rolipram (Figure 8A, *p* < 0.05) (Figure 8B). The changes of downstream protein kinase A and G (PKA and PKG) expression were also examined in our subsequent study, which suggested that baicalein treatment increased both PKA and PKG levels [Figures 8C–E, F_(5, 42)_ = 13.27, *p* < 0.05; F_(5, 42)_ = 11.34, *p* < 0.05]. However, treatment with rolipram only increased PKA level (*p* < 0.001).

Aβ induced neuronal structural and functional changes in some brain regions, such as hippocampus and frontal cortex, decrease the phosphorylation of CREB, i.e. pCREB, which may block the downstream proteins expression such as BDNF (Cui et al., 2019). As shown in Figures 8F,G, we found that treatment with Aβ42 oligomers significantly decreased ratio of pCREB to CREB in the hippocampus of mice. However, this decreased pCREB/CREB ratio in the hippocampus was significantly reversed by baicalein, when compared to vehicle-treated Aβ42 oligomers groups [Figure 8E, F_(5, 42)_ = 12.58, *p* < 0.05]. Given that the primary role of BDNF is to promote the functional architecture of neurons in the brain (Cho et al., 2013), our further study determined BDNF expression in the hippocampus of Aβ42 oligomers-treated mice after treatment with baicalein. As shown in Figures 8F,H, BDNF levels were decreased in the hippocampus of Aβ42 oligomers-treated mice, as compared to the vehicle-treated normal controls (*p* < 0.001). However, these effects were significantly rescued by either baicalein or rolipram, which suggest that the effects of the two drugs are involved in the regulation of PDE2A and PDE4 subtypes related neuroprotective response.

## Discussion

The present study showed that treatment of baicalein for 14 days rescued Aβ-induced memory and cognitive deficits in novel object recognition, Morris water maze, and shuttle-box tests. Morphological analysis revealed that baicalein rescued Aβ-induced dendritic atrophy, as evidenced by increases in the total of dendritic length, number of dendrites, and spine density. Our further study suggested that Aβ-induced decreased expression in two synapse associated proteins, i.e., synaptophysin and PSD95, were increased after treatment with baicalein. Moreover, expression of PDE2A, PDE4A and PDE4D was increased in Aβ-treated mice, which were reversed by baicalein treatment, but have no changes after rolipram treatment. Aβ-induced decreases in neuroprotective proteins, such as pCREB/CREB and BDNF, were also reversed by baicalein. These effects were similar to those of the PDE4 inhibitor rolipram. These findings suggest that baicalein protects neurons against Aβ insults by regulation of PDE2A/PDE4 subtypes and synapse-related cAMP/cGMP-CREB-BDNF signaling pathway.

Baicalein (5,6,7-trihydroxyflavone; C_15_H_10_O_5_; the chemical structure in Figure 1A) is an important flavonoid component in the roots of Scutellaria baicalensis Georgi (Labiatae) (Sowndhararajan et al., 2017). Previous study suggested that baicalein exhibits wide range of pharmacological activities including antioxidant, anti-inflammatory, anticancer, antiviral and neuroprotective properties (Dinda et al., 2017). Recent studies have showed that baicalein has anti-convulsive and anxiolytic effects. It also protects neurons against excitatory neurotransmitters such as glutamate and neurotoxic substances, for example, it protects against amyloid-β (Aβ), 6-hydroxydopamine (6-OHDA), and methamphetamine induced neuronal cell lesion both *in vitro* and *in vivo* (Gasiorowski et al., 2011; Yu et al., 2012; Wei et al., 2014). These findings sufficiently support baicalein as promising resource for the development of a natural neuroprotective agent. However, how and whether effects of baicalein on Aβ-induced cognitive deficits are related to neuronal remodeling related PDE2- and PDE4-cAMP/cGMP signaling are still unknown.

The novel object recognition (NOR) paradigm utilizes the natural tendency of rodents to explore novel objects in order to reflect an implicit form of episodic memory (Ennaceur & Delacour, 1988; Xu et al., 2015). This is usually used for examining the early stages of cognitive deficits in animal models of Alzheimer’s disease. In this study, We observed that Aβ-treated mice showed impairment of memory consolidation and retrieval processes in the NOR test. Baicalein treatment produced memory-enhancing effects, evidenced by mice spending more time exploring the novel objects 24 h after training. These results are agree with the subsequent studies from Morris water maze and shuttle-box tests. In the MWM test, Aβ exposure resulted in impaired acquisition, as mice treatment with Aβ showed longer latency to find the platform in the training session (learning curve). In probe trials, the number of crossings and the latency to touch the previous platform location were increased as compared to those of control groups, suggesting impaired memory consolidation and retrieval. However, these mice that were treated with baicalein showed ameliorated acquisition ability, i.e. learned faster in the training sessions, and restored memory retrieval. Similarly, baicalein reversed Aβ-induced increase in latency to escape to avoid foot shock, further supporting that baicalein ameliorated memory and cognitive impairment induced by neurotoxic substances such as Aβ. Indeed, these 3 behavioral tests represent memory performance in different stages of AD. For example, NOR examines the sporadic memory representative of early stage of memory deficits in AD. The MWM and shuttle-box usually assess the spatial and aversive memory processes representative of mid- and late-stages of memory deficits. Critically, baicalein rescue of Aβ-induced sporadic, spatial and aversive memory deficits indicates that baicalein is effective in AD-related cognitive impairment and dementias.

Neuronal atrophy is one of the most significant pathological changes in the AD brain (Oboudiyat et al., 2013). Aβ may decrease neuronal sprouting and growth through its deleterious effects on the CNS function (Mrdjen et al., 2019). In this study, we found that chronic treatment with baicalein reversed Aβ-induced decreases in the dendric length, number of dendrites and spine density in the CA1 of hippocampus, suggesting that baicalein could rescue Aβ-induced deleterious effects. These findings consistent with the previous studies, which suggested that flavonoids protect neurons against neurotoxic substance induced neuronal atrophy and cell death (Rajput et al., 2017; Ardah et al., 2020). Moreover, our study found that the expression of presynaptic and postsynaptic proteins, i.e., synaptophysin and PSD95, was decreased in the Aβ-treated group. This decrease was rescued by treatment with baicalein, further supporting that baicalein ameliorates memory and cognitive function by regulation of neuroplasticity and the related synapse associated proteins.

Recent studies suggest that baicalein protects neurons against Aβ-induced memory impairment by regulation of neuroinflammation and cell apoptosis (Gu et al., 2016; Li et al., 2019). Up to now, no evidence has suggested whether these effects are related to PDEs activity and the related pathway. Our studies along with others demonstrate that the high levels of PDE2, PDE4 and its subtypes were found in Aβ-treated mice hippocampi (Xu et al., 2015; Wang et al., 2017; Cui et al., 2019). Previous studies suggested that chronic treatment with PDE2 or PDE4 inhibitors may enhance memory via amelioration of neuronal remodeling in the frontal cortex and hippocampus, regions vulnerable to Aβ insults (Zhang et al., 2018; Ruan et al., 2019). The improvement of neuroplasticity in the Aβ-treated mouse brain after baicalein treatment was significant in our present study, which may be related to inhibition of PDE2A, PDE4A, 4B and PDE4D expression in the hippocampus. Indeed, some studies suggest that the expression of PDEs subtypes is decreased or even lost from cortico-cortical synapses and dendrites with advanced age in primates and rats (Carlyle et al., 2014; Leslie et al., 2020), which indicates that inhibition of PDEs’ activities may be associated with impaired rather than improved cognitive performance. Recently, controversy has increased over different expression of PDE subtypes and their activities in different brain regions with aging (Tibbo et al., 2019). We noticed that increasing studies support that PDE4 subtypes’ level may vary in isoform-specific manner when mice are chronically treated with PDEs inhibitors (Zhang & O'Donnell, 2000; Miro et al., 2002). These results demonstrate that adaptive alterations in PDE2A and PDE4 subtypes may occur in a chronic dosing protocol. In the present study, we found that levels of PDE2A, PDE4A, 4B and PDE4D were increased in Aβ42 oligomers-treated mouse hippocampus, which were reversed by chronic treatment with baicalein for 14 days. It is possible that baicalein-induced anti-Aβ effects may mainly occur through regulation of PDE2A, PDE4A, PDE4B and PDE4D related signaling. Further study suggested that both cAMP and cGMP levels were increased after chronic treatment with baicalein, supporting the amelioration of baicalein on memory function and neuronal remodeling may be via PDE2A and PDE4 subtypes dependent cAMP/cGMP pathway.

It is well known that the upregulation of cAMP and cGMP levels is usually associated with an increase in PKA/PKG-related CREB phosphorylation. Ser133 is one of three potential phosphorylation sites in human CREB protein which contributes to regulation of neurodegenerative disorders and memory impairment (Xia et al., 2019). The present study found that Aβ exposure caused significant reduction in pCREB at Ser133, evidenced by a decreased ratio of pCREB/CREB. Baicalein prevented this decreased ratio, which indicates that baicalein may rescue cAMP/cGM-PKA/PKG dependent CREB phosphorylation and its downstream signaling. Recent studies have demonstrated that molecular determinants of neuronal plasticity, such as pCREB and BDNF, are activated by neuroprotective therapy, resulting in improved memory and cognitive function (Wang et al., 2016; Wang et al., 2017; Cocco et al., 2020). Our study suggested that baicalein significantly rescued Aβ-induced BDNF expression reduction, indicating that such effects may be related to regulation of baicalein on PDE2A/PDE4-dependent neuroprotective pathway that ultimately improves neuronal remodeling and cognitive function.

In conclusion, baicalein ameliorates memory impairment and neuronal atrophy induced by microinjection of Aβ into CA1 of hippocampus of mice. By modeling of Aβ-induced neurotoxicity in mice, baicalein triggers PDE2A/PDE4-related neuroplasticity and neuroprotective pathway by the rescue of synapse associated proteins, i.e. synaptophysin and PSD95, and cAMP/cGMP-related CREB phosphorylation and neurotrophic factor BDNF expression.

## Data Availability

The raw data supporting the conclusion of this article will be made available by the authors, without undue reservation.
